# Impact of COVID-19 Pandemic Lockdown on the Prognosis, Morbidity, and Mortality of Patients Undergoing Elective and Emergency Abdominal Surgery: A Retrospective Cohort Study in a Tertiary Center, Saudi Arabia

**DOI:** 10.3390/ijerph192315660

**Published:** 2022-11-25

**Authors:** Rakan H. Alelyani, Ali H. Alghamdi, Saad M. Mahrous, Bader M. Alamri, Mudhawi H. Alhiniah, Maisa S. Abduh, Saleh M. Aldaqal

**Affiliations:** 1 College of Medicine, Faculty of Medicine, King Abdulaziz University, Jeddah 21589, Saudi Arabia; 2 Immune Responses in Different Diseases Research Group, King Abdulaziz University, Jeddah 21589, Saudi Arabia; 3 Department of Medical Laboratory Sciences, Faculty of Applied Medical Sciences, King Abdulaziz University, Jeddah 21589, Saudi Arabia; 4 Department of Surgery, Faculty of Medicine, King Abdulaziz University, Jeddah 21589, Saudi Arabia

**Keywords:** COVID-19, SARS-CoV-2, abdominal surgery, Saudi Arabia

## Abstract

The SARS-CoV-2 pandemic’s main concerns are limiting the spread of infectious diseases and upgrading the delivery of health services, infrastructure, and therapeutic provision. The goal of this retrospective cohort study was to evaluate the emergency experience and delay of elective abdominal surgical intervention at King Abdul-Aziz University Hospital from October 2019 to October 2020, with a focus on post-operative morbidity and mortality before and during the COVID-19 pandemic. This study compares two groups of patients with emergent and elective abdominal surgical procedures between two different periods; the population was divided into two groups: the control group, which included 403 surgical patients, and the lockdown group, which included 253 surgical patients. During the lockdown, surgical activity was reduced by 37.2% (*p* = 0.014), and patients were more likely to require reoperations and blood transfusions during or after surgery (*p*= 0.002, 0.021, and 0.018, respectively). During the lockdown period, the average length of stay increased from 3.43 to 5.83 days (*p* = 0.002), and the patients who developed complications (53.9%) were more than those in the control period (46.1%) (*p* = 0.001). Our tertiary teaching hospital observed a significant decline in the overall number of surgeries performed during the COVID-19 pandemic and lockdown period. During the lockdown, abdominal surgery was performed only on four patients; they were positive for COVID-19. Three of them underwent exploratory laparotomy; two of the three developed shock post-operative; one patient had colon cancer (ASA score 3), one had colon disease (ASA score 2), and two had perforated bowels (ASA scores 2 and 4, respectively). Two out of four deaths occurred after surgery. Our results showed the impact of the COVID-19 lockdown on surgical care as both 30-day mortality and total morbidity have risen considerably.

## 1. Introduction

The first pandemic of the new century was brought on by the unique virus known as the severe acute respiratory syndrome coronavirus (SARS-CoV) [1]. In December 2019, pneumonia cases with an unknown cause were reported in China’s Wuhan City [2]. Subsequently, the World Health Organization (WHO) deemed COVID-19 a public health emergency of international concern in January 2020 [2]. On 2 March 2020, Saudi Arabia reported its first incidence of COVID-19, and on 3 April 2020, the country went into full lockdown [3,4]. Unusual circumstances, such as the recent COVID-19 pandemic, put a great deal of pressure on medical professionals to alter hospital infrastructure and protocols to stop the spread of contagious viruses and ensure the efficient delivery of medical care [5]. An area of health care that requires serious policy alterations is the surgical care services, one of the most significant health care units. Intensive care ability needs to be increased, needs the conversion of recovery rooms and even waiting rooms into intensive care beds [6]. In such cases, hospital resources, such as personal protective equipment (PPE), ventilators, and transfusion supplies, should be reserved for COVID-19 patients [6,7].

As a result of the COVID-19 pandemic, several organizations have developed new guidelines that instruct surgeons on what procedures to perform [7,8]. Only emergency, cardiovascular, and oncological procedures were conducted in this critical circumstance, which worsened the problem of non-COVID-19 patients’ delayed emergency arrangement [9,10]. Studies showed that there were few emergency admissions and more individuals delayed getting treatment during the pandemic [11,12]. This delayed presentation should be considered a significant healthcare issue because it could result in a worse prognosis. In COVID-19 patients, emergency surgery is linked to worse outcomes [13]. The concerns can significantly rise when considering old and fragile patients, who often develop COVID-19 infection. When COVID-19 is present, the mortality rate can reach 40%, as opposed to 23% in the same population without COVID-19 [13,14].

Contrary to elective treatments, which may typically be postponed with only minimal risk to patients, delaying final surgical therapy for acute surgical procedures can result in a significant increase in morbidity and mortality rates [15,16]. The general postponement of elective and urgent surgery could make patients more susceptible to adverse consequences [17]. There is an urgent need for information on the treatment and prognosis of surgical patients with COVID-19, and recent recommendations suggest against non-delayable operations [18,19].

Acute surgical care must continue as the scientific community continues to investigate the illness process and its consequences for certain systems. In addition, it is crucial to find how the COVID-19 pandemic would affect post-operative outcomes in both emergency and elective surgery. Spain, Italy, and other countries affected by the COVID-19 pandemic reported a decrease in acute care surgical activity (ACSA) [15,16].

As a result, we designed this study to evaluate and present our experience with an emergency, postponed elective abdominal surgical intervention at King Abdul-Aziz University Hospital from October 2019 to October 2020, with a focus on post-operative morbidity and mortality before and during the COVID-19 pandemic.

## 2. Materials and Methods

### 2.1. Patients and Study Design

This study is a retrospective cohort study comparing two distinct groups of patients undergoing emergent and elective abdominal surgical procedures between two different time periods. The control group (six months before the lockdown date) ranges from 1 October 2019 to 3 April 2020, and the lockdown (pandemic) group ranges from 4 April 2020 to 4 October 2020. The data of all patients were collected with confidentiality at King Abdul-Aziz University Hospital, Jeddah, using the hospital information system (Phoenix). In the current study, all patients who underwent for urgent or scheduled abdominal surgery were included. Patients under the age of 18 and cases that do not need an abdominal surgical intervention, pregnant women, trauma cases, and bariatric surgeries were excluded from this study.

### 2.2. Variables and Outcome

Characteristics and demographic data extracted from the hospital database including age, gender, body mass index (BMI), smoker or non-smoker, diagnosis, and comorbidity (e.g., diabetes mellitus, hypertension, lung disease, autoimmune disease, kidney disease, immunodeficiency status, anemia, hemophilia, liver disease, neurological disorders, cancer, thyroid disease, and cardiac disease). The BMI range for underweight category <18.5 and for healthy weight is 18.5 to 24.9 kg/m^2^. A BMI of 25 to 29.9 kg/m^2^ is regarded as overweight, and a BMI of 30 kg/m^2^ or more is considered obese [20].

The status of COVID-19-positive patients, the date of operations, antibiotics, and deep venous thrombosis (DVT) prophylaxis, any prophylactic dose of heparin, low molecular weight heparin, unfractionated heparin, and directed oral anticoagulation the status of surgery (elective or emergent), re-operation (if re-operation occurs in 30 days post-operative), the name of the operation, and intraoperative and post-operative blood transfusion were investigated.

Furthermore, according to the American Society of Anesthesiologists Classification (ASAC), surgical duration (minutes), the wound infection category [21], complications, and whether the patient was discharged or not were evaluated. Patients who were not discharged were those who required an ICU stay, mechanical ventilation, or who passed away. Complications include those post-operative complications, such as neurological, urinary, wound, respiratory, GI, electrolyte abnormalities, shock, sepsis, venous thromboembolism, and arrhythmia. Surgical wound classification is an evaluation of how contaminated a surgical wound was at the time of the operation. There are four types of wound classifications: clean, clean-contaminated, contaminated, and dirty or infected [22]. The duration of post-operative follow-up was 30 days. We had four patients with positive COVID-19 who underwent surgical intervention. We only presented our experience, and we did not apply any statistical relationship because their number is so low that a conclusion about their outcome cannot be drawn.

The primary outcome is to assess the post-operative morbidity and mortality of abdominal surgical procedures in the two periods. The Clavien-Dindo classification has five grades. Grade 1 includes any deviation from the normal post-operative course without the need for pharmacological treatment or surgical, endoscopic, or radiological interventions. Grade 2 includes any patient who requires pharmacological treatment with drugs other than those allowed for grade 1 complications. Blood transfusions and total parenteral nutrition are also included. Grade 3 includes any patient who requires surgical, endoscopic, or radiological intervention. Grade 4 includes any patient who requires ICU management. Grade 5 includes the death of a patient [23].

### 2.3. Ethical Statement

This study was approved by the research ethics committee at KAUH under reference 42/22. There was no requirement for formal informed consent; however, all medical history, clinical, and laboratory data, including the patient’s information, was anonymized to guarantee that only anonymous data were analyzed.

### 2.4. Statistical Analysis

In the present study, data were presented as mean ± standard deviation (SD), and the statistical analysis was conducted using the Statistical Package for the Social Sciences (SPSS) program version 21 to assess and evaluate the hypothesis. Categorical variables were compared using the chi-square test. Additionally, an independent *t*-test was applied to check if there was a significant difference between the two groups. Binomial logistic regression was used to evaluate the predictors of the binary outcome variable. The cut-off value for significance was set at *p* < 0.05.

## 3. Results

### 3.1. Basic Demographic Characteristics of Patients

A complete shutdown was declared by the Saudi government on 3 April 2020, because of COVID-19 dissemination. This study included a total of 656 patients who underwent abdominal surgical operations between 1 October 2019 and 4 October 2020. In the present study, the population was divided into two groups: the control group (6 months before the lockdown date), which included 403 surgical patients, and the lockdown group (6 months after the lockdown date), which included 253 surgical patients.

The basic demographic characteristics of patients are summarized in Table 1. The mean age of the control group was 43.7 ± 16.03, with 189 (46.9%) males and 214 (53.1%) females, while the mean age for the lockdown group was 44.5 ± 16.89, with 114 (45.1%) males and 139 (54.9%) females. When comparing the control period to the lockdown period, statistically significant increases in BMI (obesity) of patients or those with a history of smoking were noticed with *p* values of 0.005 and 0.018, respectively. The ASA score system showed a statistically significant increase in the control group compared to the lockdown group *p* = 0.019. On the other hand, no significant differences were detected in age (*p* = 0.531), sex distribution (*p* = 0.646), nationality (*p* = 0.775), those with a history of chronic anticoagulation (*p* = 0.188), or immunosuppressive use (*p* = 0.382) in the two periods.

**Figure 1:**
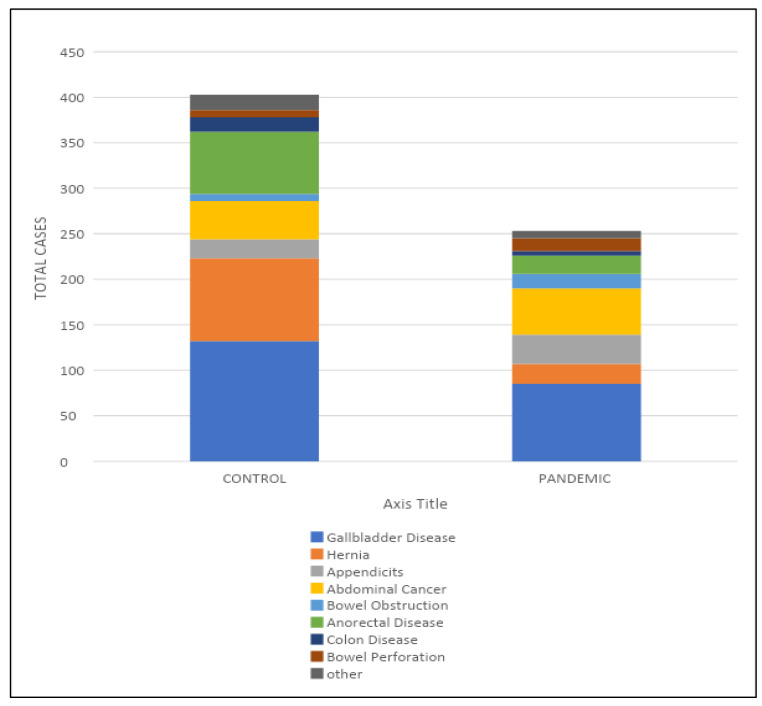
Types of surgeries performed during control and lockdown periods.

Figure 1 demonstrates that a statistically significant reduction (37.2%) was observed in the total number of surgically diagnosed and treated patients in the control group in comparison with the lockdown group (*p* = 0.014).

### 3.2. Assessment of the Peri-Operative Variables and Surgical Activities

Table 2 shows the pre-operative assessment and surgical activities of patients during the two periods. It was observed that the proportion of patients with gallbladder disease, appendicitis, abdominal cancer, bowel blockage, and bowel perforation in the lockdown group compared to the control group had a statistically significant increase (*p* = 0.001). Whereas the proportion of patients with a hernia, anorectal disease, colon disease, or other diagnosis presented during the control period exhibited a statistically significant increase compared with the lockdown group (*p* = 0.001). For patients receiving DVT prophylaxis or not, no statistically significant differences were shown (*p* = 0.601). Infection category showed a statistically significant increase in the control group compared to the lockdown group *p* < 0.001.

In addition, the average duration of an operation in the lockdown group was found to be 166.8 min as opposed to 133.7 min in the control group (*p* = 0.002). Moreover, 62.4% of all surgical operations during the lockdown were emergencies, versus 19.1% in control (*p* < 0.001). Patients were more likely to require intraoperative or post-operative blood transfusions and to be re-operated during the lockdown than they were during the control period (*p* = 0.021, *p* = 0.018, and *p* = 0.002, respectively).

### 3.3. Clinical and Surgical Consequences of Patients

According to the clinical outcome presented in Table 3, the mean length of stay in the hospital was found to have increased from 3.43 to 5.83 days in the lockdown period compared with the control period (*p* = 0.002). A total of 89 patients (41 patients in the control period and 48 patients during the lockdown period) were observed to develop complications during the studied period, with a statistically significant increase in the lockdown (18.9%) compared to the control group (10.1%) (*p* = 0.001). Mortality was higher in the lockdown group (21.7%) compared to the control group (15.3%) with *p* = 0.039). The Clavien-Dindo classification identified 44.6% of control patients as grade 4 versus 26.96% of lockdown patients (*p* = 0.003). In this study, 273 patients in the control group and 75 in the lockdown group were not assessed by the Clavien-Dindo classification as they did not fulfil the criteria for this classification.

### 3.4. Profile of COVID-19 Patients Who Underwent Surgical Procedures

During the lockdown, abdominal surgery was performed on four people (3 males and 1 female) who had positive COVID-19 PCR results and normal BMI values (average ranging from 16.78 to 24.45). Two of the four patients were Saudi nationals and two were not; their average ages ranged from 26 to 86 years. Three of the four patients had comorbid illnesses and suffered from different consequences, such as shock and electrolyte disturbance; all four patients fell under the category of contaminated infections. One of the four patients had colon cancer (ASA score 3), one had colon disease (ASA score 2), and two had perforated bowels (ASA scores 2 and 4, respectively), and the surgeries for the patients lasted for 209, 305, 112, and 153 min, respectively. One received a hemicolectomy, and the other three underwent exploratory laparotomies. During the procedures, blood was transfused into three of them. However, two developed bowel perforations and required an emergency exploratory laparotomy; they were in severe condition. After surgery, two out of four patients passed away.

### 3.5. Relationships between Complications and Their Risk Variables

Table 4 shows the relationships between complications occurring during the lockdown period and their risk variables, including socio-demographic information, general health status, and surgery details, using a univariate regression analysis test. In Table 4, male, underweight, and gallbladder disease were used as references in comparisons of variables such as sex, BMI, and diagnosis, respectively.

Some relative risk factors were significantly linked to increased complications in the current investigation, including age at diagnosis, female sex, abdominal cancer, bowel perforation, colon disease, and bowel obstruction. Cancer, cardiac disease, chronic immunosuppression, chronic anticoagulation, reoperation, blood transfusion intraoperatively, post-operatively, or for the duration of surgery were also statistically associated with complications. Additionally, there was a statistically significant correlation between the complications and other relative risk variables in the infection category, including contamination, dirt, length of stay, ASAC class 2, 3, and 4, or more. Conversely, other risk factors, such as being healthy, overweight or obese, smoking, having a hernia, appendicitis, and anorectal disease, did not exhibit any statistically significant correlation with the complications.

## 4. Discussion

Since 2019, COVID-19, a global pandemic disease, has had an influence on human health services all over the world [2,3]. WHO declared COVID-19 to be a public health emergency of global concern at the beginning of 2020 [2]. The spread of COVID-19 had a detrimental effect on the economy and healthcare system in several nations, including Saudi Arabia [2]. On 3 April 2020, the Saudi government declared a state of lockdown. As a result of COVID-19, 4898 people died and 336,766 became infected on 5 October 2020, according to statistics from the Ministry of Health of Saudi Arabia. Accordingly, during the pandemic, international societies advised postponing avoidable surgery, both oncological and urgent, due to several hospitals collapsing [24,25,26]. Concerns have been raised that COVID-19 would cause delays in elective surgery by deferring non-urgent procedures. The benefit of surgery declines as waiting times increase since it has been demonstrated that delays in surgery have an impact on results, with longer waits to lead to a worse prognosis in many diseases [27,28,29]. Individuals with SARS-CoV-2 infection and conditions that are frequently treated surgically, such as acute cholecystitis or appendicitis, were urged to receive antibiotic treatment before undergoing urgent surgeries due to the urgency of the situation [30,30].

In addition, studies have indicated higher mortality and complications suffered by patients undergoing surgery during the pandemic, particularly those with a SARS-CoV-2 infection [31,32]. Furthermore, it was reported that some patients avoided visiting the hospital unless it was an emergency because they feared contracting COVID-19 [30,31]. The need for this group was motivated by widespread concern among surgical patients about the unanticipated consequences of the SARS-CoV-2 lockdown, which had an impact on morbidity and mortality rates [33,34].

One of the objectives of this study was to compare the clinical and surgical outcomes of the two groups (control and lockdown) and investigate how COVID-19 affected both emergency and elective surgical procedures. It is one of the few studies that were conducted in Saudi Arabia involving the initial phase of the COVID-19 pandemic. The outcome of this study is in line with a study conducted in England, which reported that female involvement during control and lockdown periods was remarkably high [35].

The results of this study revealed that Saudi patients underwent surgery frequently during both time periods because of easier access to public hospitals compared with non-Saudi patients. Along with a study conducted in France, it was proportionally observed that obesity was higher in the control period compared to the lockdown period, when a healthier lifestyle predominated [36].

Furthermore, it was noted that there were more non-smokers in the lockdown group compared to the control group, which is consistent with the results of the earlier study conducted in 61 nations worldwide [37]. Contrary to the findings of a study conducted in England, which showed that colon diseases were more common, and gallbladder diseases were more frequently operated on in both control and lockdown periods in this study [35]. This difference might be explained by Saudi Arabia’s gallbladder disease incidence, which is typically high [36]. In line with international findings, there was a 37.2% decrease in surgical activity during the lockdown (*p* = 0.014) [38,39,40]. Due to a decrease in elective surgeries performed (from 80.89% to 37.5%), there were more cases of bowel perforation, appendicitis, and abdominal cancer during the lockdown period. Surgical activities have been affected by Saudi Arabia’s announcement of a national lockdown, and some patients were reluctant to visit hospitals during the pandemic [41]. In both groups, ASA class 2 was more prevalent, which is consistent with a recent study conducted in tertiary care hospitals in India [42]. While other research by Chonlada et al., 2021 found no variation in the length of the operation between the two periods, the average surgical duration in this study was found to have increased significantly from (133.7) to (166.8) minutes during the lockdown period (*p* = 0.002) [43].

The surgical wound infection category was classified mainly into clean or contaminated between control and lockdown periods, mainly due to the process that demonstrates pre-operative COVID-19 swab testing, a PPE dressing, and undressing guide, restrictions on medical staff in the operating room, the necessity to take a shower after taking off PPE, and the necessity to keep the operating room door closed at all times during surgery [44]. The average length of hospital stays during the control and lockdown periods were found to be 3.43 and 5.83 days, respectively. This is lower than the length of hospital stays under lockdown reported in a previous study conducted in an Indian tertiary care facility, which was 10.7 days [42]. The Clavien-Dindo classification during the control period was mainly grade 4, unlike the lockdown period, in which grade 1 was predominant. However, grades 0–2 were dominated in both control and lockdown periods in Italy [45]. Only 32.2% of patients in the control group were classified, whereas 70.3% of patients in the lockdown group deviated from the normal post-operative course and were classified by the Clavien-Dindo-classification. This could be due to the different guidelines across countries. This study was unable to differentiate between COVID-19 infections that occurred before and during hospital inpatient admission. Organizations, such as the American College of Surgeons and the Centers for Disease Control and Prevention in the United States, recommend negative pressure operating rooms for patients who test positive for or are suspected of having COVID-19 infection [46]. Researchers who have written academic publications agree with this viewpoint because, during a routine laparoscopy, pneumoperitoneum leakage could expose the surgical team to aerosols [47,48]. We have noted that surgeries were restricted to emergency cases and that elective cases were delayed. Accordingly, the number of patients and the complications increased.

According to De Simone et al., laparoscopy in COVID-19 patients should ideally be avoided, especially in emergencies [49]. The United Kingdom’s Royal College of Surgeons recommended only using minimally invasive surgery in carefully considered individual cases where the clinical benefits outweighed the potential risk of virus transmission [18,49]. At the same time, an earlier study found that previous SARS-CoV-2 infection was linked to a higher risk of post-operative pulmonary problems (10.7% vs. 3.6%), a higher rate of post-operative complications (58% vs. 6%), and a higher mortality rate [50].

We have encountered four COVID-19 patients in our study (3 males and 1 female) with positive COVID-19 PCR, three of them have healthy BMI, and one is underweight. They underwent abdominal surgery during the lockdown period. All four of the patients came into the category of contaminated infections; three of the patients had comorbidities conditions and encountered complications (Shock, Electrolytes disturbance); two of the patients died after surgery. The four patients’ average ages ranged from 26 to 86 years, two of them were Saudi nationals and two were not. One of the four patients had colon cancer, one had colon illness, and two of the patients had perforated bowels. Three out of four were subject to a blood transfusion during the operations and two of them have a post-operative blood transfusion as well. Out of the four, three underwent exploratory laparotomies and the fourth underwent a hemicolectomy. On the other hand, two were in critical condition; they had bowel perforation and underwent emergency exploratory laparotomy, they have ASA scores of 2 and 4, and the duration of surgery was 112 and 153 min, respectively.

These findings emphasize the potential impact of COVID-19 on surgical patients with variable severity, but no inferences can be drawn in this regard due to the small number of COVID-19 patients in our sample. Indeed, clinical and statistical factors may have contributed to complications without affecting the death rate. Some limitations were found in this study. This study was single-center research and there may be variations when compared to different locations or nations. Since most patients were in emergent cases during the lockdown, the COVID-19 status in this study was unreliable; a more exact status update is needed to further support the outcomes of COVID-19 and non-COVID-19 patients. This study was conducted on patients at a tertiary teaching facility. As management standards and patient outcomes are not uniformly excellent throughout Saudi Arabia, our findings might only signify a higher standard of medical treatment than those found in other research in the same area. Consequently, a comprehensive study on the effects of the COVID-19 pandemic on the prognosis, morbidity, and mortality of patients receiving acute abdominal surgery, involving numerous hospitals and medical centers from different Saudi Arabian provinces, is crucial.

## 5. Conclusions

In the spring and fall of 2020, during the COVID-19 pandemic and lockdown period, our tertiary teaching hospital experienced a decline in the overall number of surgical procedures, but a rise in the ratio of emergency surgical operations was observed. The results of this study demonstrated how COVID-19 affected surgical care, increasing morbidity rates due to delayed hospital access and widespread COVID-19 infection concerns. To prove that the COVID-19 epidemic has an impact on surgical management and outcomes, more research is required. The global community will be better able to distinguish between patients who might benefit from non-operative therapy and be free of complications, and those who should be referred to surgical departments as soon as possible.

## Figures and Tables

**Table 1:** Basic demographic characteristics of patients during the pre-COVID (control group) and COVID periods (Lockdown group).

Characteristic	Control	Lockdown	*p*-Value
	(*n* = 403)	(*n* = 253)	
Age * (Mean ± SD)	43.7 ± 16.03	44.5 ± 16.89	0.531
Gender ** (Number and %)			0.646
Male	189 (46.9%)	114 (45.1%)	
Female	214 (53.1%)	139 (54.9%)	
Nationality **			0.775
Saudi	297 (73.7%)	189 (74.7%)	
Non-Saudi	106 (26.3%)	64 (25.3%)	
BMI **			0.005
Underweight	18 (4.5%)	14 (5.57%)	
Healthy	104 (26%)	89 (35.45%)	
Overweight	127 (31.75%)	85 (33.8%)	
Obese	154 (38.21%)	63 (25.1%)	
Smoker **	33 (8.19%)	9 (3.55%)	0.018
Non-Smoker	370 (91.81%)	244 (96.45%)	
ASA Score **			0.019
Class 1	157 (39.05%)	76 (30.0%)	
Class 2	182 (45.3%)	115 (45.45%)	
Class 3	57 (14.16%)	56 (22.1%)	
Class 4 or more	7 (1.49%)	6 (2.27%)	
Chronic anticoagulant use **	15 (3.72%)	15 (5.92%)	0.188
Immunosuppression therapy use **	29 (7.19%)	23 (9.09%)	0.382

BMI, body mass index. * Independent t-test was conducted at *p* value = 0.05, ** *p* Value was calculated by person chi-square test.

**Table 2:** Assessment of the peri-operative variables and surgical activities of the control and lockdown groups.

Characteristic	Control	Lockdown	*p*-Value
	(*n* = 403)	(*n* = 253)	
Diagnosis **			0.001
Gallbladder disease	132 (32.75%)	85 (33.6%)	
Hernia	91 (22.58%)	22 (8.7%)	
Appendicitis	21 (5.21%)	32 (12.6%)	
Abdominal cancer	42 (10.42%)	51 (20.2%)	
Bowel obstruction	8 (1.98%)	16 (6.3%)	
Anorectal disease	68 (16.87%)	20 (7.9%)	
Colon disease	16 (3.97%)	5 (1.98%)	
Bowel perforation	8 (1.98%)	14 (5.53%)	
Others	17 (4.2%)	8 (3.1%)	
DVT prophylaxis use **	166 (41.19%)	99 (39.1%)	0.601
Duration of surgery * (minutes)	133.7 (120.32)	166.8 (135.10)	0.002
Mean (SD)			
Status of surgery **			<0.001
Elective	326 (80.89%)	95 (37.5%)	
emergency	77 (19.1%)	158 (62.4%)	
Re-operation **	3 (0.74%)	11 (4.34%)	0.002
Intra-operative blood transfusion **	33 (8.18%)	35 (13.8%)	0.021
Post-operative blood transfusion **	17 (4.21%)	22 (8.7%)	0.018
Infection category **			<0.001
Clean	80 (21.85%)	18 (7.1%)	
Clean/contaminated	179 (45%)	130 (51.4%)	
Contaminated	127 (31.9%)	96 (37.9%)	
Dirty	7 (1.25%)	9 (3.6%)	

DVT, deep vein thrombosis; ASAC, American Society of Anesthesiologists Classification. * *p*-value was calculated by *t*-test, ** *p* Value was calculated by person chi-square.

**Table 3:** Clinical and surgical consequences of patients during control and lockdown periods.

Characteristic	Control	Lockdown	*p*-Value
	(*n* = 403)	(*n* = 253)	
Length of stay *	3.43 (8.78)	5.83 (10.21)	0.002
Mean (SD)			
Clavien-Dindo Classification **			0.003
Grade 1	54 (41.5%)	88 (49.4%)	
Grade 2	11 (8.46%)	17 (9.55%)	
Grade 3	2 (1.53%)	18 (10.11%)	
Grade 4	58 (44.6%)	48 (26.96%)	
Grade 5	5(3.84%)	7 (3.93%)	
Discharge **	341 (84.7%)	198 (78.3%)	0.039
Not-discharge	62 (15.3%)	55 (21.7%)	
Complication **	41 (10.1%)	48 (18.9%)	0.001

* *p*-Value was calculated by *t*-test, ** *p*-Value was calculated by person chi-square.

**Table 4:** Binary logistic regression (Univariate) of patients who developed post-operative complications during the lockdown. (*n* = 48).

Risk Factors		*p*-Value	OR	95% C.I. for OR		Nagelkerke R Square
				Lower	Upper	
Age at Diagnosis		0.005	1.027	1.008	1.047	0.051
Sex	Male		Ref.			
	Female	0.019	0.464	0.244	0.882	0.035
BMI	Underweight (<18.5 kg/m^2^)		Ref.			0.018
	Healthy (18.5–24.9 kg/m^2^)	0.204	0.456	0.136	1.529	
	Overweight (25–29.9 kg/m^2^)	0.128	0.386	0.113	1.316	
	Obese (≥30 kg/m^2^)	0.100	0.340	0.094	1.228	
Smoker	Yes	0.800	1.230	0.247	6.115	0.000
Diagnosis	Gallbladder disease		Ref.			0.360
	Hernia	0.288	2.733	0.428	17.466	
	Appendicitis	0.522	1.822	0.290	11.444	
	Abdominal cancer	0.001	12.495	3.422	45.623	
	Bowel obstruction	0.001	27.333	6.023	124.042	
	Anorectal disease	0.758	1.439	0.142	14.603	
	Colon disease	0.001	109.333	9.195	1299.994	
	Bowel perforation	0.001	49.200	10.051	240.836	
	Other	0.003	16.400	2.611	102.997	
Known case of cancer	Yes	0.002	4.232	1.706	10.498	0.056
Cardiac disease	Yes	0.046	2.746	1.019	7.399	0.023
Chronic immunosuppressive therapy	Yes	0.003	3.887	1.588	9.510	0.051
Chronic anticoagulation	Yes	0.001	7.654	2.577	22.731	0.083
Status of surgery	Elective					0.012
	Emergency	0.185	1.586	0.802	3.139	
Reoperation	Yes	0.001	23.423	4.873	112.589	0.132
Blood transfusion intraoperatively	Yes	0.001	17.636	7.689	40.452	0.294
Blood transfusion post-operative	Yes	0.001	16.583	6.041	45.522	0.203
Duration of surgery (minutes)		0.001	1.005	1.003	1.007	0.121
Infection category	Clean		Ref.			0.211
	Clean/contaminated	0.829	1.264	0.151	10.613	
	Contaminated	0.034	9.323	1.189	73.123	
	Dirty	0.034	13.600	1.225	151.045	
LOS		0.001	1.332	1.219	1.456	0.496
ASAC	Class 1		Ref.			0.251
	Class 2	0.026	4.221	1.192	14.944	
	Class 3	0.001	16.960	4.756	60.477	
	Class 4 or more	0.001	121.667	10.628	1392.816	

OR, Odd ratio; BMI, body mass index; ASAC, American Society of Anesthesiologists Classification; and LOS, length of stay.

## Data Availability

The data presented in this study are available on request from the corresponding author. The data are not publicly available due to [privacy].
